# WHO declares end of mpox global health emergency: first glance from a perspective of ChatGPT/GPT-4

**DOI:** 10.1097/JS9.0000000000000543

**Published:** 2023-06-19

**Authors:** Yanqiu Lu, Shaoyan Qi, Kunming Cheng, Haiyang Wu

**Affiliations:** a Department of Intensive Care Unit, The Second Affiliated Hospital of Zhengzhou University, Zhengzhou, Henan; b Department of Graduate School, Tianjin Medical University, Tianjin, People’s Republic of China; c Duke Molecular Physiology Institute, Duke University School of Medicine, Durham, North Carolina, USA

*Dear Editor*, Mpox is an emerging zoonotic infectious disease caused by the mpox virus, an orthopoxvirus and close relative of the variola virus. Since early May 2022, it has received much attention due to the reports of multiple mpox cases not only in countries where the disease is endemic but also in countries where it is typically not found^1,2^. With the increased number of confirmed cases, on 23 July 2022, the World Health Organization (WHO) declared mpox outbreak a Public Health Emergency of International Concern (PHEIC). According to WHO data, since the outbreak of the mpox epidemic, a total of 111 countries and regions have reported over 87 000 cases to the WHO, including 140 fatalities. However, as the number of confirmed cases gradually decreased, the WHO announced on 11 May that mpox was no longer a PHEIC. In our previous bibliometric study, we found that mpox-related research is the most neglected branch among the four orthopoxvirus genera^3,4^. Thus, we called for more research groups devoted to this area. According to data from the PubMed database, more than 2000 studies have been published in the recent 2 years. These data suggest that national governments and international organizations have increased their investment in this field. However, as the mpox PHEIC was finally declared over, many researchers and academics are concerned that the investments and resources for tackling mpox would dwindle, and efforts triggered by the 2022 outbreak would not be sustained^5^.

In view of this, at the time point of WHO announcing the end of mpox PHEIC, our group conducted an online survey by using the extremely popular artificial intelligence (AI)-based chatbot called ChatGPT (GPT-4, https://chat.openai.com/chat). The aim of the present investigation was to summarize opinions from the perspective of ChatGPT/GPT-4. As shown in Additional file 1 (Supplemental Digital Content 1, http://links.lww.com/JS9/A722), four questions including the basic knowledge of PHEIC, underlying reasons for the end, long-term mpox management and continual monitoring plan, as well as the potential role of ChatGPT/GPT-4 for monitoring mpox in the future, were put forward by us.

According to the answer from GPT-4, the decision to end the PHEIC status is mainly related to the following aspects such as decrease in the number of new cases, effective control measures, and reduced risk of international spread. This is indeed the case, according to the data from WHO, the number of reported cases in the past 3 months has decreased by nearly 90% compared to the preceding 3 months. During the mpox PHEIC, a large number of studies on vaccines, testing, and treatments have been performed worldwide^6–8^. This could be an important reason for such rapid control of mpox. Meanwhile, the end of PHEIC does not mean that the epidemic threat completely disappears. This implies that the prevention and control efforts in various countries have taken on a new phase. From a long-term perspective, GPT-4 has proposed multiple recommendations including continual monitoring and surveillance, further research, strengthening public health infrastructure, vaccine development, public education, maintaining strong international cooperation, and regular risk assessments (Fig. 1). These suggestions could serve as an important reference for developing a long-term response plan for mpox. Of note, recently, on 5 May 2023, WHO also announced the end of coronavirus disease 2019 (COVID-19) PHEIC and propose a long-term COVID-19 management plan including 5 core components of COVID-19 preparedness, readiness and response, and 10 operational pillars. However, such a long-term management plan for mpox is still missing. Therefore, we suggest that drawing up such an official long-term management plan should not be neglected.

**Figure 1:**
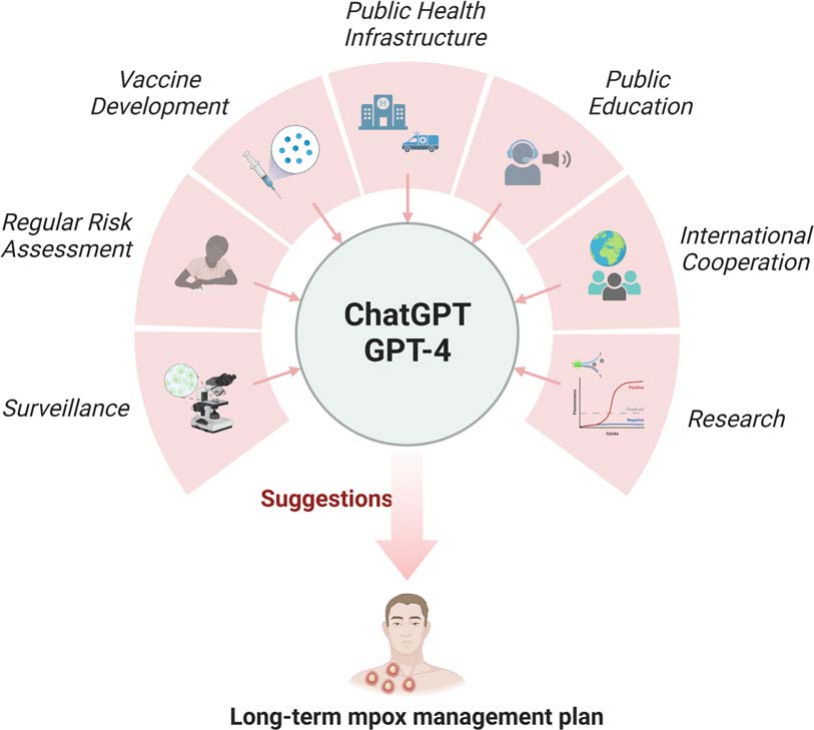
Long-term mpox management plan from ChatGPT/GPT-4 (created with BioRender.com).

In addition, ongoing advancements in AI technologies have been implemented in numerous fields. For instance, regarding AI and machine learning, a large body of previous research has been performed in the field of infectious diseases, especially for COVID-19^9^. During the COVID-19 pandemic, many kinds of chatbots for psychological support, telemedicine, health education, and so on^10,11^. Multiple previous studies also have discussed the potential applications of ChatGPT/GPT-4 in infectious diseases^12,13^. In this study, we further explored the potential role of AI-based chatbots like ChatGPT/GPT-4 for monitoring mpox outbreaks in the future. As can be seen from the answers of GPT-4, this program could an important role in several ways including real-time information analysis, predictive modeling, public engagement, assisting health professionals, data interpretation, and language translation. Take public engagement as an example, during the mpox and COVID-19 epidemic, many studies reported the anxiety and depression disorders of medical staff and ordinary people due to concerns over one’s risk of contracting these infectious diseases^14^. Chatbots like ChatGPT/GPT-4 could help the public to acquire authoritative health information and provide emotional support. More research is needed to explore the specific role of AI in this field.

All in all, although the mpox PHEIC was finally declared over, it continues to spread around the world and the threat of its resurgence remains. Thus, a long-term management plan for this disease has only begun. As we have suggested, drawing up an official long-term management plan for mpox is critically important. Moreover, the introduction of AI like ChatGPT/GPT-4 into clinical practice is now an unstoppable process and will play a significant role in future emerging infectious diseases.

## Ethical approval

This study does not include any individual-level data and thus does not require any ethical approval.

## Sources of funding

None.

## Author contribution

Y.L.: methodology, formal analysis, investigation, and writing – original draft; S.Q.: methodology, data curation, formal analysis, and resources; K.C.: conceptualization, methodology, data curation, resources, and investigation; H.W.: conceptualization, formal analysis, and resources.

## Conflicts of interest disclosure

The authors declare no conflicts of interest.

## Research registration unique identifying number (UIN)

1. Name of the registry: not applicable.
2. Unique identifying number or registration ID: not applicable.
3. Hyperlink to your specific registration (must be publicly accessible and will be checked): not applicable.

## Guarantor

Haiyang Wu.

## Data availability statement

The data underlying this article will be shared by the corresponding author upon reasonable request.

## Supplementary Material

SUPPLEMENTARY MATERIAL
