# Supplementary material for: WHO declares end of mpox global health emergency: first glance from a perspective of ChatGPT/GPT-4
Source: Int J Surg. 2023 Jun 19;109(10):3217–8. doi: 10.1097/JS9.0000000000000543 (PMC10583947; doi:10.1097/JS9.0000000000000543)
Supplement: SUPPLEMENTARY MATERIAL [file js9-109-3217-s001.docx]

1. **Do you know what is a Public Health Emergency of International Concern (PHEIC)?**

GPT-4: *Yes, a Public Health Emergency of International Concern (PHEIC) is a formal declaration by the World Health Organization (WHO). It is used to announce that an extraordinary event is taking place which is determined to: Constitute a public health risk to other states through the international spread of disease, and potentially require a coordinated international response.*

*This concept and its regulations are set out in the 2005 International Health Regulations (IHR), which is a legally binding agreement involving 196 countries across the globe, including all the member states of WHO. Declaring a PHEIC is meant to mobilize international attention and resources to address the public health situation. Some notable examples of PHEICs include the H1N1 (swine flu) pandemic in 2009, the Ebola virus disease outbreak in West Africa in 2014, the Zika virus outbreak in 2016, and the COVID-19 pandemic in 2020.*

1. **On July 23, 2022, the World Health Organization (WHO) declared mpox outbreak a Public Health Emergency of International Concern (PHEIC). As the number of confirmed cases gradually decreased, the WHO announced on 11 May that mpox was no longer a PHEIC. What do you think of the end of mpox PHEIC and the underlying reasons?**

GPT-4: *The World Health Organization (WHO) declaring the end of a Public Health Emergency of International Concern (PHEIC) for the monkeypox (mpox) outbreak indicates that the situation has significantly improved since the initial declaration. The decision to end the PHEIC status is usually guided by specific criteria, including: (1) The decrease in the number of new cases: If there has been a steady decline in the number of new mpox cases, it suggests that the outbreak is being effectively controlled. (2) Effective control measures: If the affected countries and international partners have put in place effective measures to control the spread of the disease, and these measures appear to be working, this would support the decision to end the PHEIC. (3) The reduced risk of international spread: If the risk of the disease spreading to other countries has been significantly reduced, this would also justify ending the PHEIC.*

*The decision to declare an end to a PHEIC would be based on a careful assessment of the available evidence, and it would likely have been made in consultation with an Emergency Committee of experts. It's an encouraging sign that the situation has improved, but it doesn't mean that efforts to control the disease can be relaxed. Ongoing surveillance, control measures, and vigilance will be necessary to prevent a resurgence of the disease.*

1. **As the WHO announced mpox was no longer a PHEIC. What are your suggestions for a long-term mpox management and continual monitoring plan?**

GPT-4: *After the World Health Organization (WHO) has declared that the monkeypox (mpox) outbreak is no longer a Public Health Emergency of International Concern (PHEIC), there are several measures that can be taken for long-term management and monitoring of the disease. It’s crucial to remember that although the immediate crisis might have passed, ongoing efforts are needed to prevent future outbreaks and minimize the impact of the disease. Here are some suggestions: (1) Surveillance: Continual monitoring and surveillance of monkeypox cases should be maintained. This includes reporting and tracking of all cases, including mild ones. Rapid detection and reporting can help control potential future outbreaks. (2) Research: Further research should be conducted on the virus to understand its biology, ecology, and transmission better. This includes research into potential animal reservoirs and modes of human-to-human transmission. (3) Public Health Infrastructure: Strengthen public health infrastructure in areas where monkeypox is prevalent. This can involve improving diagnostic capabilities, increasing access to healthcare, and training health workers to recognize and respond to monkeypox cases. (4) Vaccine Development: If a vaccine for monkeypox does not exist, efforts should be directed towards its development. If a vaccine is available, strategies should be made to ensure its distribution, especially in endemic areas. (5) Public Education: Educate the public about monkeypox, its symptoms, how it's transmitted, and what to do if they suspect they or someone else has it. This can help in early detection and treatment, as well as preventing the spread of the disease. (6) International Cooperation: Maintain strong international cooperation and communication. Share data and research findings with the global community to enhance the collective understanding and response to monkeypox. (7) Regular Risk Assessment: Conduct regular risk assessments to identify any changes in the epidemiology of the disease that could indicate an increased risk of an outbreak.*

*By implementing these measures, it’s possible to maintain control over monkeypox, even though it's no longer classified as a PHEIC. However, it’s crucial to remain vigilant and flexible, as the situation can change.*

1. **The potential role of ChatGPT/GPT-4 for monitoring mpox outbreaks in the future.**

GPT-4: *GPT-4 or similar AI models like ChatGPT can play several roles in monitoring and managing future outbreaks of diseases like monkeypox. It’s important to remember that AI models aren’t a replacement for human experts or established systems, but they can complement and enhance these systems in several ways: (1) Real-time Information Analysis: AI models can analyze vast amounts of data quickly, making them valuable for real-time surveillance. They can process and analyze news articles, social media posts, and other data sources to detect early signs of an outbreak. (2) Predictive Modeling: AI models can use available data to create predictive models that can help anticipate the spread of the disease, identify high-risk areas, and guide public health interventions. (3) Public Engagement: AI chatbots can be used to disseminate accurate information about monkeypox to the public, answer questions, and dispel myths or misinformation. This could be particularly valuable during an outbreak, when there might be a lot of fear and confusion. (4) Assisting Health Professionals: AI can assist health professionals by providing quick access to the latest research, guidelines, and data about monkeypox. This can support decision-making and help ensure that interventions are based on the most current evidence. (5) Data Interpretation: AI models can also help in the interpretation of complex data, making it easier for public health officials and other stakeholders to understand the current situation and make informed decisions. (6) Language Translation: AI models are capable of translating content between different languages, which can be particularly useful in global health contexts, where information needs to be shared across countries and cultures.*

*However, there are also some important limitations and challenges to consider. AI models depend on the availability and quality of data. They can’t create information; they can only analyze what’s available. They also can’t replace the expertise and judgement of public health professionals. Furthermore, there are ethical and privacy considerations when using AI in health contexts, especially when it involves personal health data. Finally, AI models like GPT-4 can generate outputs based on their training data up until a certain date (September 2021 for GPT-4), and can’t access or process information about events in the world that occurred after this date.*
